# A prospective cohort study of *Plasmodium falciparum* malaria in three sites of Western Kenya

**DOI:** 10.1186/s13071-022-05503-4

**Published:** 2022-11-09

**Authors:** Benyl M. Ondeto, Xiaoming Wang, Harrysone Atieli, Daibin Zhong, Guofa Zhou, Ming-Chieh Lee, Pauline Winnie Orondo, Kevin O. Ochwedo, Collince J. Omondi, Simon M. Muriu, David O. Odongo, Horace Ochanda, James Kazura, Andrew K. Githeko, Guiyun Yan

**Affiliations:** 1 Department of Biology, University of Nairobi, Nairobi, 00100 Kenya; 2 Program in Public Health, College of Health Sciences, University of California at Irvine, Irvine, CA 92697 USA; 3 Sub-Saharan Africa International Center of Excellence for Malaria Research, Tom Mboya University, Homa Bay, 40300 Kenya; 4 Department of Biochemistry, Jomo Kenyatta University of Agriculture and Technology, Nairobi, 00200 Kenya; 5 Department of Biological Sciences, Pwani University, Kilifi, 80108 Kenya; 6 Center for Global Health and Disease, Case Western Reserve University, Cleveland, OH 44106 USA; 7 Centre for Global Health Research, Kenya Medical Research Institute, Kisumu, 40100 Kenya

**Keywords:** Malaria, Transmission, Resurgence, *Plasmodium falciparum*

## Abstract

**Background:**

Malaria in western Kenya is currently characterized by sustained high Plasmodial transmission and infection resurgence, despite positive responses in some areas following intensified malaria control interventions since 2006. This study aimed to evaluate long-term changes in malaria transmission profiles and to assess patterns of asymptomatic malaria infections in school children aged 5–15 years at three sites in western Kenya with heterogeneous malaria transmission and simultaneous malaria control interventions.

**Methods:**

The study was conducted from 2018 to 2019 and is based on data taken every third year from 2005 to 2014 during a longitudinal parasitological and mosquito adult surveillance and malaria control programme that was initiated in 2002 in the villages of Kombewa, Iguhu, and Marani. *Plasmodium* spp. infections were determined using microscopy. Mosquito samples were identified to species and host blood meal source and sporozoite infections were assayed using polymerase chain reaction.

**Results:**

*Plasmodium falciparum* was the only malaria parasite evaluated during this study (2018–2019). Asymptomatic malaria parasite prevalence in school children decreased in all sites from 2005 to 2008. However, since 2011, parasite prevalence has resurged by > 40% in Kombewa and Marani. Malaria vector densities showed similar reductions from 2005 to 2008 in all sites, rose steadily until 2014, and decreased again. Overall, Kombewa had a higher risk of infection compared to Iguhu (*χ*^2^ = 552.52, *df* = 1, *P* < 0.0001) and Marani (*χ*^2^ = 1127.99, *df* = 1, *P* < 0.0001). There was a significant difference in probability of non-infection during malaria episodes (log-rank test, *χ*^2^ = 617.59, *df* = 2, *P* < 0.0001) in the study sites, with Kombewa having the least median time of non-infection during malaria episodes. Gender bias toward males in infection was observed (*χ*^2^ = 27.17, *df* = 1, *P* < 0.0001). The annual entomological inoculation rates were 5.12, 3.65, and 0.50 infective bites/person/year at Kombewa, Iguhu, and Marani, respectively, during 2018 to 2019.

**Conclusions:**

Malaria prevalence in western Kenya remains high and has resurged in some sites despite continuous intervention efforts. Targeting malaria interventions to those with asymptomatic infections who serve as human reservoirs might decrease malaria transmission and prevent resurgences. Longitudinal monitoring enables detection of changes in parasitological and entomological profiles and provides core baseline data for the evaluation of vector interventions and guidance for future planning of malaria control.

**Graphical abstract:**

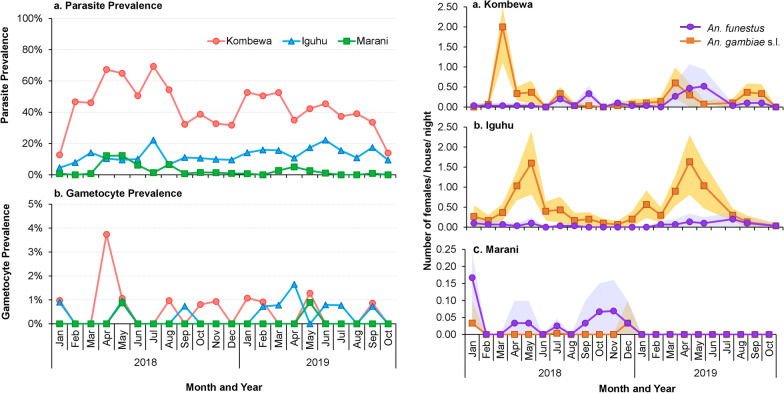

**Supplementary Information:**

The online version contains supplementary material available at 10.1186/s13071-022-05503-4.

## Background

Globally, an estimated 241 million cases of malaria were reported in 2020 resulting in approximately 627,000 deaths; 96% of these deaths occurred in Africa and children aged < 5 years accounted for 77% of these deaths [1]. In Kenya, an estimated 27 million malaria cases and 12,600 deaths attributed to malaria were reported in 2020 [1]. Since 2000, malaria mortality and morbidity have declined significantly in African countries, including Kenya, and have been attributed chiefly to the scale-up of insecticide-treated net (ITN) distributions, indoor residual spraying (IRS), and artemisinin-based combination therapies (ACTs) [2]. Nonetheless, malaria remains a major public health concern in Africa.

Kenya’s Ministry of Health began the country’s first free mass long-lasting insecticidal net (LLIN) distribution in 2006 to children under 5 years and pregnant women, followed by a second distribution in 2011 aiming for universal coverage in targeted areas [3]. Thereafter, there have been three successive rounds of distribution in 2014, 2017, and 2021 to boost LLIN coverage and replace worn nets [3–5]. Indoor residual spraying applications began in 2005 to prevent epidemics in malaria epidemic-prone areas in the highlands [6]. To reduce the malaria burden in the Lake Victoria endemic zone, IRS was implemented in targeted districts from 2008 to 2012 [6–8]. However, IRS was not applied from 2012 to 2016 because of the detection of widespread pyrethroid resistance in malaria vector populations and lack of a registered non-pyrethroid insecticide in the country [9, 10]. After 5 years of no treatments, IRS was restarted in 2017 with the micro-encapsulated organophosphate insecticide pirimiphos-methyl (Actellic® 300CS) and applied during successive rounds from 2018 to 2021 in two targeted counties (Migori and Homa Bay) located in the Lake Victoria endemic zone, where intense malaria transmission occurs throughout the year [11]. Artemisinin-based combination therapies began in 2004 after several years of sulfadoxine-pyrimethamine treatments (1998–2003) and earlier recognition of widespread antimalarial drug failures (e.g. chloroquine) [12, 13]. Malaria control programmes face numerous challenges, among them development of pyrethroid resistance in malaria vectors [14], changes in vector dominance and behaviour [15–17], and the emergence of antimalarial drug resistance [18]. In an effort to mitigate insecticide resistance, the World Health Organization (WHO) has recommended conducting IRS with organophosphate and neonicotinoid insecticides and using pyrethroid-piperonyl butoxide (PBO) synergized treated nets [1], which have been distributed in targeted counties in Kenya from 2020 to 2021.

Despite these malaria control efforts, areas in western Kenya are experiencing heterogeneity in malaria transmission after interventions, with some areas indicating a decline in transmission, while in others, transmission has remained unchanged or has resurged [5, 19–21]. A study in western Kenya linked these contrasting outcomes to malaria vector species composition shifts, insecticide resistance, and climatic warming [21]. Similar observations of varying outcomes in malaria control have been observed elsewhere in Africa [22].

This study aimed to evaluate long-term changes in malaria transmission profiles and patterns of asymptomatic malaria infection in three sites with different transmission intensities in western Kenya after distributions of new pyrethroid-PBO treated LLINs and applications of new IRS formulations. Hopefully, the results presented here will help in assessing vector interventions, serve as a baseline for the evaluation of new interventions, and guide future control planning by the Kenya National Malaria Control Programme.

## Methods

### Study site

**Fig. 1:**
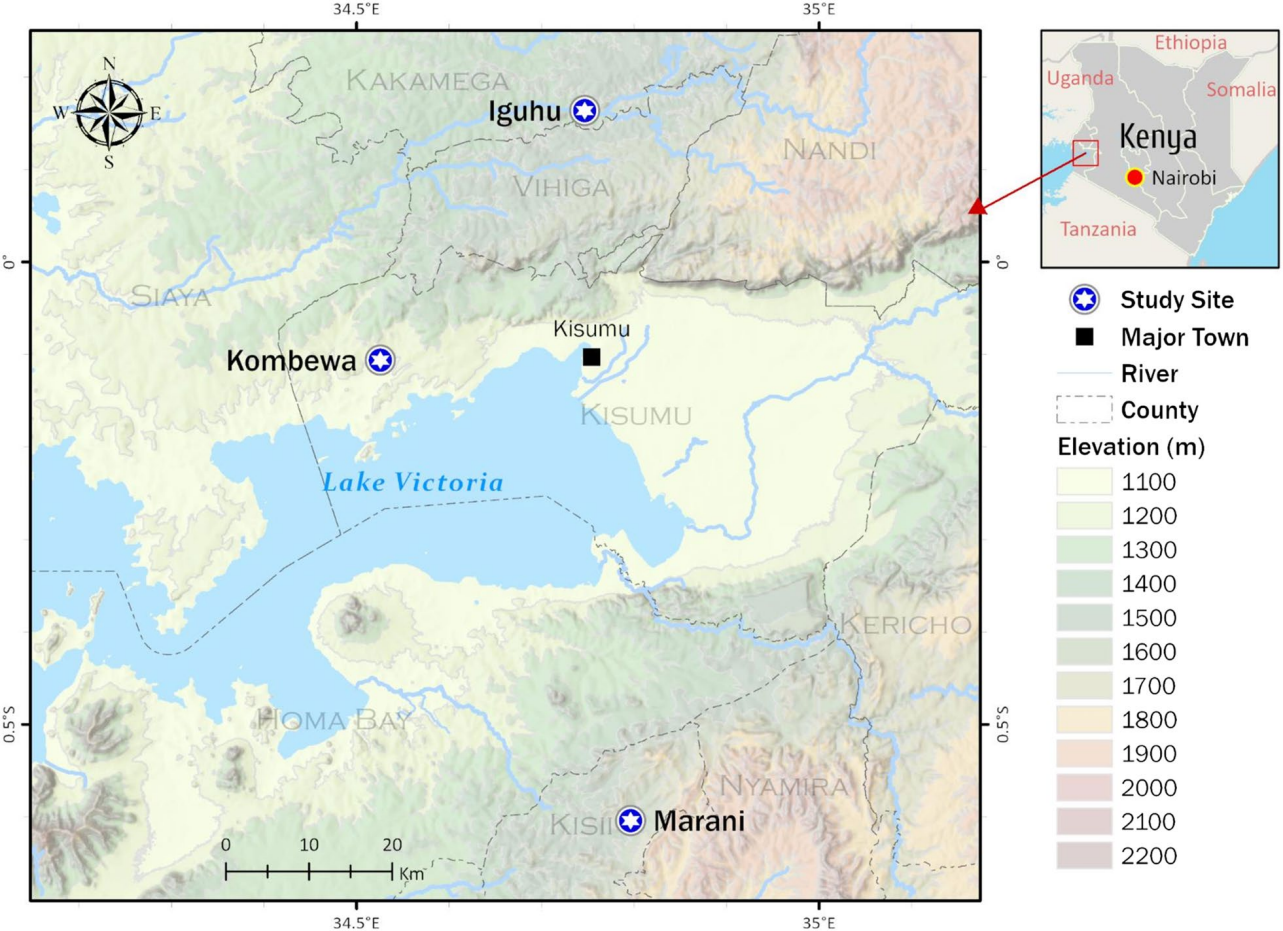
Map of the study sites in western Kenya

The study was conducted in three sites in western Kenya, each with different malaria transmission intensity: two highland sites, Iguhu (0°08′53′′N; 34°47′16′′E, 1430–1580 m elevation) (mesoendemic) in Kakamega County and Marani (0°35′13′′S; 34°48′11′′E, 1540–1740 m elevation) (hypoendemic) in Kisii County, and one lowland site in Kombewa (0°07′10′′S; 34°29′04′′E, 1150–1300 m elevation) (holoendemic) in Kisumu County (Fig. 1).

The climate in western Kenya consists mainly of a bimodal pattern of rainfall, a long rainy season between April and June, and a short rainy season between October and November [19]. The hot and dry season is from January to February while the cool and dry season from July to September [19]. All sites have shown variations in monthly cumulative precipitation and monthly mean maximum and minimum temperatures, ranging from 29.1 °C to 14.5 °C, respectively [19, 21, 23].

*Plasmodium falciparum* is the primary malaria parasite species in the three sites [19]. The first mass distribution of LLINS in 2006 in western Kenya led to a decline of both asymptomatic malaria and clinical cases [21]. The second mass distribution in 2011 was characterized by a positive response at Iguhu but Kombewa and Marani experienced sustained high *P*. *falciparum* transmission and infection resurgences, respectively, despite a third round of LLIN distributions in 2015 [21].

The predominant malaria vector species in the study sites are *Anopheles gambiae* s.s., *An*. *arabiensis*, and *An*. *funestus* [19, 24]. In the lowland site, *An*. *funestus* is the most abundant and infectious malaria vector, while in the highland sites *An*. *gambiae* s.s. is the main vector responsible for Plasmodial transmission. Recent studies in this region have observed an increase in the proportion of *An*. *arabiensis* in the highlands because of vector interventions using LLINs and IRS; these measures may be suppressing the more anthropophilic and endophilic *An*. *gambiae* s.s. and killing fewer of the more zoophilic *An*. *arabiensis* [25]. Hence, high bednet coverage in western Kenya may explain decreases in vector densities of *An. gambiae* s.s. in the three sites, reductions of *An. funestus* in Iguhu and Kombewa, and temporal alterations in feeding behaviour of *An. gambiae* to earlier host seeking [20].

### Study design

#### Historic *Plasmodium falciparum* parasite prevalence and vector densities

This study was based on longitudinal parasitological and adult vector surveillance that commenced in 2002 (Iguhu) and 2003 (Kombewa and Marani) [19] to date. Snapshots of these data were taken every 3 years from 2005 to 2014 [5, 20, 21]. Data (years 2005, 2008, 2011, and 2014) from this period form the basis for the current study conducted between 2018 and 2019.

#### Parasitological surveys

**Fig. 2:**
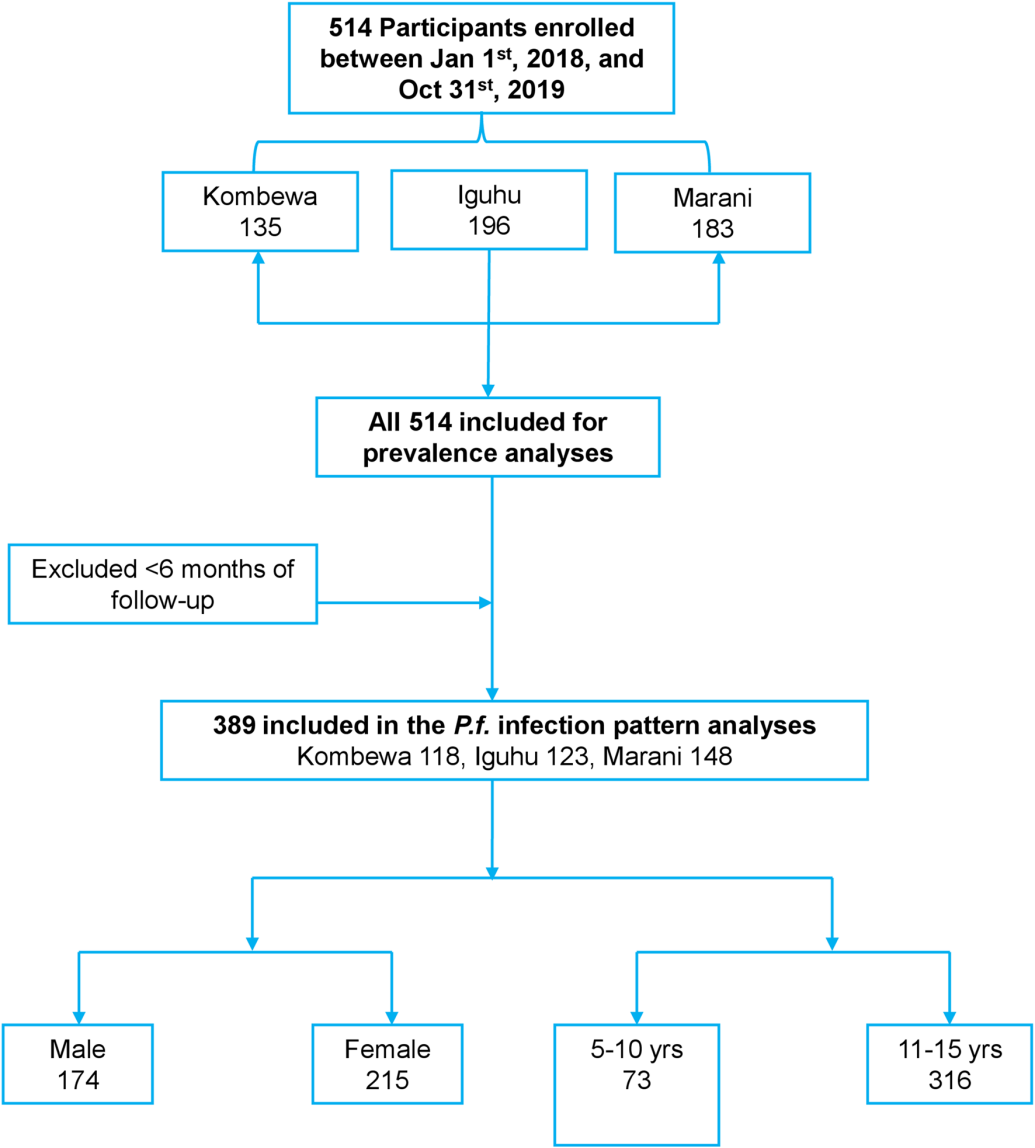
Study design flow chart of the cohort study

A cohort of 514 volunteer school-aged children aged 5–15 years were enrolled (January–March 2018) for monthly *Plasmodium* spp. surveys between 1 January 2018 and 31 October 2019 in Kombewa, Iguhu, and Marani (Fig. 2). The sample size was calculated based on the size of the study population and parasite prevalence from a previous study [5]. Consent was obtained from parents or guardians before children could participate in the study. Children with no reported chronic or acute illness, except malaria, were allowed to participate in the study. At the sampling time, children who were found to have fever were referred to the nearest government health facility for diagnosis and treatment according to Kenyan government malaria treatment guidelines [26].

Blood samples were collected using the finger-prick method and thick and thin smears prepared on labeled slides for malaria parasite species identification and parasite counts using microscopy. Malaria parasite counts were scored against 200 leukocytes. A second microscopist carried out random checks on the slide counts to ensure microscopy quality. Parasite density was expressed as parasites per μl, assuming a count of 8000 white blood cells per μl of blood [27]. *Plasmodium* spp. infection data collected from all participants were subjected to prevalence analyses; however, only participants with at least 6 months of follow-up were included in the *Plasmodium* spp. infection pattern analyses, including duration and probability of non-infections (Fig. 2).

#### Entomological surveys

Collections of indoor resting vector populations were conducted monthly by the pyrethrum spray catch (PSC) method [28] in 30 randomly selected houses in each study site between 1 January 2018 and 31 October 2019. Mosquitoes were identified morphologically as either *Anopheles gambiae* s.l. or *An*. *funestus* [29]. DNA was extracted [30] from the legs and wings of each mosquito specimen to speciate sibling species in *An*. *gambiae* s.l. and *An*. *funestus* using conventional polymerase chain reaction (PCR), as described by Scott et al. [31] and Koekemoer et al. [32], respectively. The DNA extracted from the abdomen of each freshly fed female mosquito was used to identify host blood meal sources using a multiplexed PCR assay [33]. The DNA extracted from the head and thorax of each mosquito specimen was used to determine sporozoite infections of *Plasmodium* spp. by using a multiplexed real-time quantitative PCR (qPCR) assay [34, 35].

### Climatic data

Mean monthly rainfall and maximum and minimum temperature from 2018 to 2019 were obtained from the Kenya Meteorological Department for meteorological stations in Kakamega (for Iguhu), Kisii (for Marani), and Kisumu (for Kombewa).

### Data management and analysis

The variations in parasite prevalence between different time periods at Kombewa, Iguhu, and Marani were compared using Tukey-Kramer HSD test of analysis of variance (ANOVA) with repeated measures. In addition, the differences of vector densities between different time periods at each site were compared using non-parametric Wilcoxon rank-sum tests. Means (95% confidence interval, CI) and proportions were calculated for vector and parasite populations. For the primary malaria species, *Plasmodium falciparum*, parasite/gametocyte prevalence for each site, each month, was expressed as the percentage of microscopically positive samples over the total number of samples tested. The Chi-square test was used to determine statistical differences in the parasite/gametocyte prevalence among the study sites and parasite prevalence by age and gender category in each study site. Geometric mean parasite density and variations in proportion by month infected in the age and gender in each site were compared using Wilcoxon/Kruskal-Wallis tests. The variations in the distribution of the proportion of surveys being infected among the study sites were determined using Tukey-Kramer HSD test of ANOVA. Multiple Imputation by Chained Equations (MICE) simulation was done to impute the missing data in the time-to-event analysis. A Kaplan-Meier curve was built to analyze the probability of non-infection during malaria episodes in each study site. The log-rank test was applied to compare the probability of non-infection during malaria episodes in the three study sites adjusted for multiple comparisons with Bonferroni corrections. Wald approximations were used for hazard ratio 95% confidence interval limit effects. Hazard ratios for the asymptomatic malaria infections were compared with proportional hazards fit by study sites, gender, and age groups.

The monthly density of adult anopheline mosquitoes in each study site was calculated as the average number of females per house per night (f/h/n) based on monthly surveys. Vector density variation among study sites was compared using Wilcoxon/Kruskal-Wallis tests. The human blood index (HBI) was calculated as the proportion of blood-fed *Anopheles* mosquito samples that had fed on humans to the total tested [36]. The sporozoite rates for each site and vector species were calculated as the proportion of *Anopheles* mosquito samples positive for *Plasmodium* spp. to the total number tested. The annual entomological inoculation rates (EIRs) for each site and vector species were calculated as the product of the sporozoite rate and human biting rates [37]. Differences in the mean annual rainfall and mean annual maximum and minimum temperatures between the study sites were computed using the Tukey-Kramer HSD test of ANOVA with repeated measures. These analyses were done using JMP Pro 16 (SAS Institute, Inc.) and R statistical software (version 4.0.3; R foundation for statistical computing, Vienna, Austria).

## Results

### Historic *Plasmodium falciparum* parasite prevalence and vector densities

Changes in parasite prevalence and vector densities in Kombewa, Iguhu, and Marani are shown in Table 1 from 2005 to 2014. Similar trends in parasite prevalence were observed in the three sites, i.e., declining parasite prevalence from 2005 to 2008 in all sites, and a rebounding trend in prevalence from 2008 in Iguhu and 2011 in Kombewa and Marani (Table 1). In Kombewa, parasite prevalence decreased slightly from 2005 (51.16%, 95% CI 46.79–55.54) to 2008 (48.06%, 95% CI 41.61–54.51) (Tukey-Kramer HSD test, *P* > 0.05) and then declined sharply from 2008 to 2011 (29.80%, 95% CI 19.50–40.10) (Tukey-Kramer HSD test, *P* = 0.006). After that, it rose significantly to 45.86% (95% CI 39.34–52.38) in 2014 (Tukey-Kramer HSD test, *P* = 0.02). In Iguhu, a sharp decline in parasite prevalence was observed from 2005 (26.61%, 95% CI 21.88–31.34) to 2008 (6.45%, 95% CI 4.58–8.32) (Tukey-Kramer HSD test, *P* < 0.0001) and rose steadily to 16.82% (95% CI 13.52–20.12) in 2014 (Tukey-Kramer HSD test, *P* = 0.002). In Marani, a steady decline of parasite prevalence was observed from 2005 (1.95%, 95% CI 0.82–3.09) to 2011 (0.35%, 95% CI 0.05–0.66) (Tukey-Kramer HSD test, *P* = 0.04), after which there was a sharp rise in 2014 (4.44%, 95% CI 3.37–5.51) (Tukey-Kramer HSD test, *P* < 0.0001).

**Table 1:** Historic *Plasmodium falciparum* parasite prevalence and vector densities in Kombewa, Iguhu, and Marani in western Kenya [Mean (95%CI)]

Study sites	Kombewa			Iguhu			Marani		
Year	Parasite prevalence (%)^a^	*An. gambiae* s.l density^b^	*An. funestus* density^b^	Parasite prevalence (%)^a^	*An. gambiae* s.l. density^b^	*An. funestus* density^b^	Parasite prevalence (%)^a^	*An. gambiae* s.l. density^b^	*An. funestus* density^b^
2005	51.16 (46.79, 55.54)	1.04 (0.14, 1.93)	2.14 (1.16, 3.12)	26.61 (21.88, 31.34)	2.56 (0.21, 4.91)	0.29 (0.09, 0.49)	1.95 (0.82, 3.09)	0.03 (0.00, 0.05)	0.00 (0.00, 0.00)
2008	48.06 (41.61, 54.51)	0.31 (0.15, 0.47)	0.52 (0.21, 0.83)	6.45 (4.58, 8.32)	0.36 (0.24, 0.48)	0.02 (0.01, 0.04)	0.43 (0.11, 0.74)	0.01 (0.00, 0.02)	0.00 (0.00, 0.00)
2011	29.80 (19.50, 40.10)	0.54 (0.37, 0.70)	0.95 (0.58, 1.32)	13.59 (9.55, 17.64)	0.37 (0.28, 0.47)	0.12 (0.04, 0.21)	0.35 (0.05, 0.66)	0.05 (0.01, 0.09)	0.29 (0.11, 0.47)
2014	45.86 (39.34, 52.38)	0.78 (0.49, 1.07)	1.38 (1.05, 1.70)	16.82 (13.52, 20.12)	0.55 (0.35, 0.75)	0.31 (0.22, 0.40)	4.44 (3.37, 5.51)	0.11 (0.03, 0.19)	0.59 (0.46, 0.71)

^a^Variations in parasite prevalence between different time periods at each study site were compared using Tukey-Kramer HSD test of analysis of variance (ANOVA) with repeated measures

^b^Variations in vector densities between different time periods at each study site were compared using non-parametric Wilcoxon rank-sum tests

The indoor resting densities of *An*. *gambiae* s.l. and *An*. *funestus* varied significantly in all sites. The vector densities showed reductions from 2005 to 2008 in all sites and thereafter rose steadily until 2014 (Table 1). Studies from 2005 and 2008 indicate that the indoor resting densities of malaria vectors decreased sharply in Kombewa from 1.04 (95% CI 0.14–1.93) to 0.31 (95% CI 0.15–0.47) f/h/n for *An*. *gambiae* s.l. (Wilcoxon test, *Z* = 1.24, *P* = 0.21) and from 2.14 (95% CI 1.16–3.12) to 0.52 (95% CI 0.21–0.83) f/h/n for *An*. *funestus* (Wilcoxon test, *Z* = 3.38, *P* = 0.0007). Similarly, a decline was observed in Iguhu with a reduction from 2.56 (95% CI 0.21–4.91) to 0.36 (95% CI 0.24–0.48) f/h/n for *An*. *gambiae* s.l. (Wilcoxon test, *Z* = 1.62, *P* = 0.11) and that of *An*. *funestus* changed significantly from 0.29 (95% CI 0.09–0.49) to 0.02 (95% CI 0.01–0.04) f/h/n (Wilcoxon test, *Z* = 4.03, *P* < 0.0001). In Marani, *An*. *gambiae* s.l. densities decreased from 0.03 (95% CI 0.00–0.05) to 0.01 (95% CI 0.00–0.02) f/h/n (Wilcoxon test, *Z* = 1.07, *P* = 0.29) between 2005 and 2008, while no *An*. *funestus* were found during the 2 years. Between 2008 and 2014, the population of indoor resting vectors rose steadily in Kombewa (*An*. *gambiae* s.l., Wilcoxon test, *Z* = 3.23, *P* = 0.001; *An*. *funestus*, Wilcoxon test, *Z* = 2.51, *P* = 0.01), Iguhu (*An*. *gambiae* s.l., Wilcoxon test, *Z* = 1.47, *P* = 0.14; *An*. *funestus*, Wilcoxon test, *Z* = 4.17, *P* < 0.0001) and Marani (*An*. *gambiae* s.l., Wilcoxon test, *Z* = 3.00, *P* = 0.003; *An*. *funestus*, Wilcoxon test, *Z* = 4.41, *P* < 0.0001).

### *Plasmodium falciparum* parasite prevalence, gametocyte prevalence, and parasite density

**Fig. 3:**
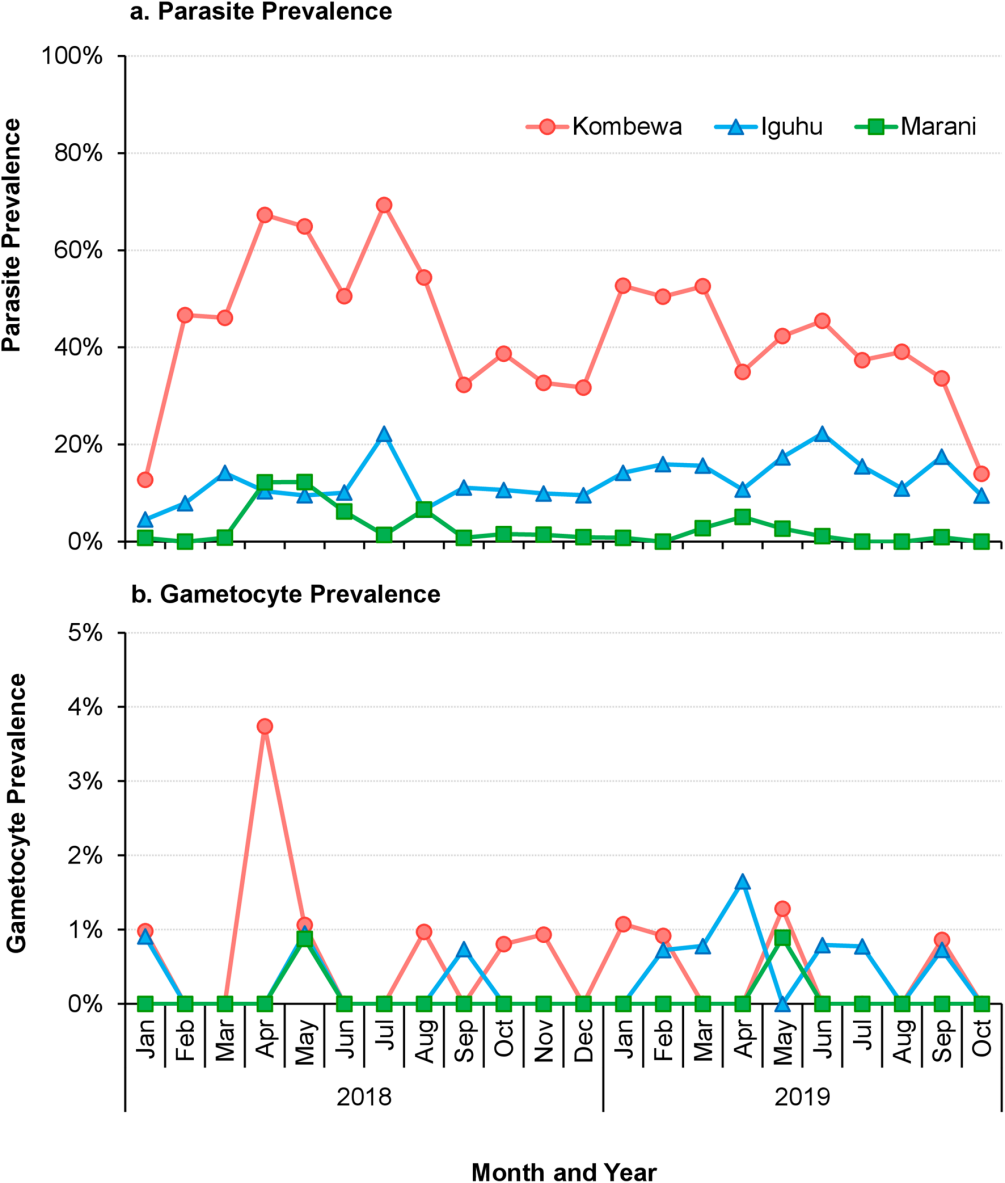
*Plasmodium* parasite prevalence (**a**) and gametocyte prevalence (**b**) in Kombewa, Iguhu, and Marani in western Kenya. Differences in the parasite/gametocyte prevalence among study sites were determined using Chi-square test

In the 2018–2019 survey, only *P*. *falciparum* was found and evaluated. The *P*. *falciparum* prevalence in Kombewa was significantly higher compared to Iguhu (*χ*^2^ = 552.52, *df* = 1, *P* < 0.0001) and Marani (*χ*^2^ = 1127.99, *df* = 1, *P* < 0.0001) (Fig. 3). Compared to 2011, parasite prevalence in 2018–2019 has resurged by > 40% in Kombewa and Marani, whereas in Iguhu, it has decreased by 7.3%. There were no significant differences in *P. falciparum* prevalence between males and females in all sites except Kombewa and no significant differences in *P. falciparum* prevalence between age groups at all sites (Additional file 3: Table S1).

The *P*. *falciparum* gametocyte prevalence was significantly higher in Kombewa compared to Iguhu and Marani (*χ*^2^ = 7.69, *df* = 2, *P* = 0.02) (Fig. 3).

In Kombewa, there was a significant difference in the geometric means of *P*. *falciparum* density between the two age groups, with higher parasite density in the 5–10 years old group. Similarly, males had higher parasite density compared to females (Additional file 3: Table S1).

### *Plasmodium falciparum* infection patterns

The proportion of months infected varied greatly in Kombewa (35.9%), Iguhu (14.9%), and Marani (5.8%) (Tukey-Kramer HSD test, *P* < 0.0001) (Additional file 1: Fig S1). No significant age and gender variations were found in the proportion of months infected in the study sites (Additional file 3: Table S1). Additional file 2: Fig S2 indicates the distribution of malaria infection patterns in the age and gender groups in the study sites.

**Fig. 4:**
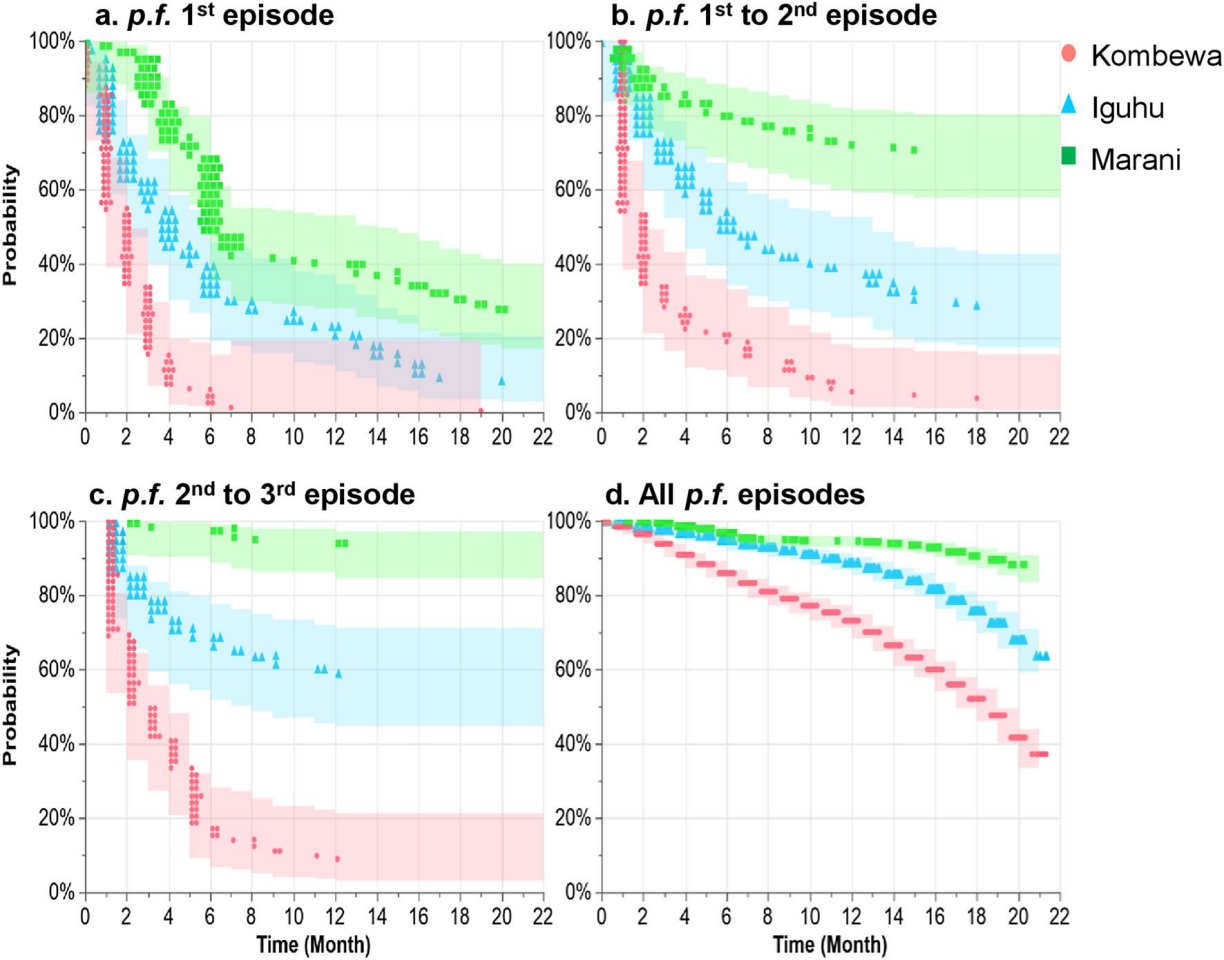
Kaplan-Meier probability of non-infection during (**a**) *p.f.* malaria first episode, (**b**) first–second episodes, (**c**) second–third episodes, and (**d**) all *p*.*f*. episodes in Kombewa, Iguhu, and Marani in western Kenya. Abbreviations: *p*.*f*., *Plasmodium falciparum*. The probability of non-infection during malaria episodes in the study sites were compared using log-rank test

As shown in Fig. 4, the median time of non-infection during malaria first episode was 1.90 [interquartile range (IQR): 1.61–2.19] months, 5.46 (IQR: 4.30–6.62) months, and 10.86 (IQR: 9.03–12.69) months in Kombewa, Iguhu, and Marani, respectively. Median time from first to second malaria episodes was 1.95 (IQR: 1.64–2.32) months, 10.37 (IQR: 8.18–12.57) months, and 65.96 (IQR: 35.38–122.98) months in Kombewa, Iguhu, and Marani, respectively. When exploring time intervals from second to third malaria episodes, the median time was 3.24 (IQR: 2.56–4.10) months, 29.10 (IQR: 16.20–52.25) months, and 491.07 (IQR: 60.23–4003.94) months in Kombewa, Iguhu, and Marani, respectively. The median time of non-infection for all malaria episodes was 17.30 (IQR: 16.77–17.75) months, 23.26 (IQR: 22.40–24.13) months, and 33.40 (IQR: 30.14–34.65) months in Kombewa, Iguhu, and Marani, respectively. There was a significant difference in probability of non-infection during malaria first episode (log-rank test, *χ*^2^ = 171.78, df = 2, *P* < 0.0001), first–second episodes (log-rank test, *χ*^2^ = 179.33, *df* = 2, *P* < 0.0001), second–third episodes (log-rank test, *χ*^2^ = 245.77, *df* = 2, *P* < 0.0001), and all episodes (log-rank test, *χ*^2^ = 617.59, *df* = 2, *P* < 0.0001) in the study sites.

For male gender, Kombewa and Iguhu sites were statistically significant risk factors associated with asymptomatic malaria infection. (Additional file 4: Table S2). Unadjusted hazard ratios for the infection were significantly higher in Kombewa and Iguhu compared to Marani, with similar results after adjustment for gender and age. Females had a significantly lower unadjusted hazard ratio for the infection than males, but was insignificant after adjustment for sites and ages.

### Vector species composition and densities

A total of 583 female anophelines were collected between 1 January 2018 and 31 October 2019, comprising 458 (78.6%) *An*. *gambiae* s.l. and 125 (21.4%) *An*. *funestus*. Of these, 479 specimens (391 *An*. *gambiae* s.l. and 88 *An*. *funestus*) were analyzed for sibling species. For the *An*. *gambiae* s.l. specimens, PCR results indicated that 77.8% were *An*. *gambiae* s.s. and 22.2% *An*. *arabiensis* in Kombewa, 85.7% *An*. *gambiae* s.s. and 14.3% *An*. *arabiensis* in Iguhu, and 33.3% *An*. *gambiae* s.s. and 66.7% *An*. *arabiensis* in Marani. All the *An*. *funestus* subjected to species identification from the study sites were confirmed as *An*. *funestus* s.s.

**Fig. 5:**
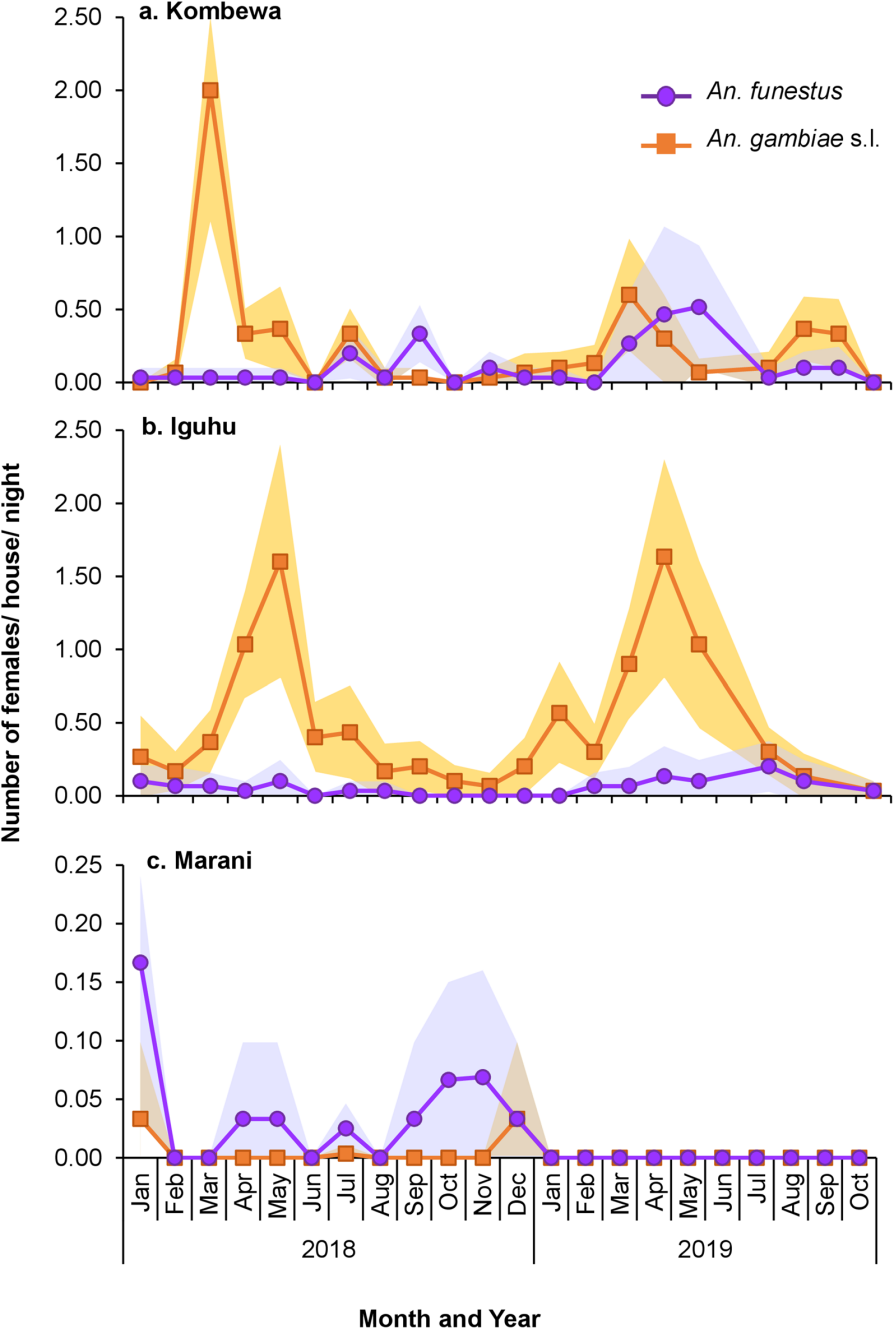
Indoor resting densities of *An. gambiae* s.l. and *An. funestus* in Kombewa (**a**), Iguhu (**b**), and Marani (**c**) in western Kenya. Differences in vector density among study sites was compared using Wilcoxon/Kruskal-Wallis tests

The mean indoor resting densities of *An*. *gambia*e s.l. were significantly different among the study sites (Wilcoxon test, *χ*^2^ = 253.44, *df* = 2, *P* < 0.0001), with Iguhu having the highest densities and Marani the lowest densities (Fig. 5; Table 2). Also, the mean densities of *An*. *funestus* were significantly different among study sites (Wilcoxon test, *χ*^2^ = 26.03, *df* = 2, *P* < 0.0001), with Kombewa having the highest densities and Marani the lowest (Fig. 5; Table 2). Compared to 2014, vector density has decreased by > 60% in all sites except in Iguhu, where *An*. *gambiae* s.l. density decreased slightly by 9%.

**Table 2:** Indoor resting densities of *An*. *gambiae* s.l. and *An*. *funestus* in Kombewa, Iguhu, and Marani in western Kenya [mean (95%CI)]

Sites	Density (female/house/night)2018		Density (female/house/night) 2019	
	*An*. *gambiae* s.l.^a^	*An*. *funestus*^a^	*An*. *gambiae* s.l.^a^	*An*. *funestus*^a^
Kombewa	0.27 (0.05, 0.49)	0.07 (0.01, 0.13)	0.24 (0.08, 0.40)	0.18 (0.00, 0.38)
Iguhu	0.42 (0.20, 0.64)	0.04 (0.00, 0.08)	0.61 (0.28, 0.94)	0.09 (0.00, 0.19)
Marani	0.00 (0.00, 0.02)	0.03 (0.01, 0.05)	0.00 (0.00, 0.00)	0.00 (0.00, 0.00)

^a^Differences in vector density among study sites was compared using Wilcoxon/Kruskal-Wallis tests

### Blood meal indices and annual entomological inoculation rate

The blood meals of *An*. *gambiae* s.l. and *An*. *funestus* were mostly of bovine (55.3%) and human (90.4%) origin, respectively, in both Kombewa and Iguhu (Additional file 5: Table S3). Due to the small number of mosquito collections in Marani, the HBI was not analyzed. Overall, the HBI of *An*. *gambiae* s.l. and *An*. *funestus* was 41.10% and 88.00%, respectively.

The annual EIR of *An*. *funestus* was threefold higher in Kombewa compared to Iguhu (Table 3). In Iguhu, the annual EIR of *An*. *gambiae* s.l. was threefold higher than the corresponding value of *An*. *funestus* (Table 3). Due to the small number of mosquito collections in Marani, the annual EIR was not analyzed. The overall total annual EIRs were 5.12, 3.65, and 0.50 infective bites/person/year (ib/p/yr) at Kombewa, Iguhu, and Marani, respectively.

**Table 3:** The entomological inoculation rate (EIR) of *Anopheles* mosquitoes in Kombewa, Iguhu, and Marani in western Kenya

Study site and species	Mean no. of sleepers/ house	Mosquito density	Sporozoite rate	HBI	Annual EIR
Kombewa					
* An*. *gambiae* s.l	2.96	0.26	0.15	0.43	2.07
* An*. *funestus*		0.12	0.24	0.86	3.05
Iguhu					
* An*. *gambiae* s.l	3.35	0.50	0.13	0.39	2.76
* An*. *funestus*		0.06	0.14	0.97	0.89
Marani					
* An*. *gambiae* s.l	3.11	0.00	0.00	1.00	0.00
* An*. *funestus*		0.02	0.44	0.50	0.50

HBI: human biting index

### Climatic data

Rainfall among the three study sites was not statistically different (ANOVA, F_(2, 69)_ = 1.24, *P* > 0.05). The mean annual maximum (ANOVA, F_(2, 69)_ = 29.72, *P* < 0.0001) and minimum (ANOVA, F_(2, 69)_ = 77.19, *P* < 0.0001) temperatures were significantly different among the sites (Additional file 6: Fig S3). The mean annual temperature between Iguhu and Marani was not significantly different (Tukey-Kramer HSD test, *P* = 0.0004), whereas it was significantly different between Iguhu and Kombewa and between Marani and Kombewa (Tukey-Kramer HSD test, all *P* < 0.0001) (Additional file 6: Figure S3).

## Discussion

This study evaluated long-term changes in malaria transmission profiles in three sites in western Kenya with heterogeneous malaria transmission and high coverage with malaria control interventions [10, 38, 39]. The study also described the pattern of asymptomatic malaria infection in the study sites. Findings of the study demonstrated that malaria prevalence remains high or has resurged in some sites despite continuous intervention efforts. Results also showed that Kombewa had a higher risk of asymptomatic infection than Iguhu and Marani and further reported a gender bias towards males in infection.

Parasite prevalence has been decreasing since 2005 in the three sites and is likely associated with a reduction in vector abundance after free mass LLIN distributions after 2006, application of IRS, and increased use of ACT treatment [8, 19]. However, there has been an observed resurgence of parasite prevalence since 2008 (Iguhu) and 2011 (Kombewa and Marani) and malaria vector densities since 2008 in all sites. These changes may be attributed to worn-out bednets and irregular use of nets; reduced optimum efficacy of LLINs over time; development of pyrethroid resistance in malaria vectors and less coverage of IRS in epidemic-prone areas [6, 19]. Additionally, in 2014 the resurgence in malaria transmission observed in Marani may also be explained by the increase in ambient temperatures between 2012 and 2015 and high rainfall in 2014 [21]. The sharp decrease in indoor resting vector densities since 2014 is likely due to continuous scaling up of LLINs in the study area. Nevertheless, despite the decrease in vector densities, persistent malaria transmission in the context of extensive malaria vector control has been observed, and this could be attributed to outdoor vector biting and resting behaviour to avoid physical contact with insecticide-treated materials, changes in vector behaviour to early evening biting and early exiting from houses, as reported in western Kenya and other parts of Africa [20, 40, 41].

The 2018–2019 study observed a higher prevalence of gametocytes in Kombewa and Iguhu than in Marani and shows that the populations living in Kombewa and Iguhu maintain a large reservoir of infectious gametocytes, thus leading to stable and continuous malaria transmission. In contrast, the population living in highland village of Marani consists of a high proportion of susceptible individuals and consequently, under suitable climatic conditions, may experience malaria resurgences [42]. Hence, monitoring air temperature and precipitation data is crucial in predicting vector and parasite dynamics, particularly in the highlands where slight changes in these parameters could lead to malaria epidemics [21].

Many factors have been associated with heterogeneity in malaria risk and include biotic, abiotic, and socio-economic factors [43]. Kombewa had the highest risk and hazard ratio of asymptomatic malaria infections in the study. Furthermore, the median time interval and probability of non-infection during malaria episodes were least in Kombewa compared to other study sites, indicating increased malaria exposure. The study further reported a gender bias towards males in asymptomatic malaria infection. Briggs et al. (2020) [44] observed that the sex-based difference might be elucidated by a slower clearance of infection in males than females due to differences in immune responses [44–46]. In other studies, this sex-based difference has been postulated to socio-behavioural factors that place men at a higher risk [47, 48]. Higher risk of malaria in male children and adolescents is likely linked to an array of physiological and behavioural changes that could contribute to the observed gender bias in this study. The possible explanations put forward for the gender difference in malaria infection include roles of sex hormones in the functioning of the immune system, immunological factors, cultural factors, and vector exposure, such as not sleeping under a net [45–47, 49]. Therefore, research studies on sex-based differences in infectious diseases such as malaria are essential for providing optimum disease management for both genders [46]. In Kombewa, young children had a higher parasite density than older individuals. The declining risk of parasitaemia as age increases has been documented in other parts of Africa with stable malaria transmission, since individuals develop semi-immunity after continued exposure to infectious mosquito bites [50, 51].

Studies conducted over 2 decades ago showed that the HBI of indoor resting *An*. *gambiae* s.s. in western Kenya was 96–97%, indicating that they had fed exclusively on humans [52, 53]. However, in this investigation, the overall HBI of *An*. *gambiae* s.l. in all study sites was only 41.1%. This behavioural plasticity in host seeking suggests that there has been a shift in blood meal sources, which could be attributed to extensive bednet coverage in the region [54]. Conversely, *An*. *funestus* was highly anthropophilic, an observation previously made in Kenya and other parts of Africa [52, 55, 56]. Furthermore, in studies conducted in Kombewa, the highly anthropophilic *An*. *funestus* has been reported to have high resistance to pyrethroids, and changes in their biting behaviour could be a major factor sustaining high transmission in the area amidst extensive malaria vector control [20, 21].

The EIRs obtained in previous studies by Githeko et al. [57] and Beier et al. [58] were exceedingly high (91–416 ib/p/yr) in western Kenya. Since then, there has been a decline in the annual *P*. *falciparum* inoculation rates, as observed by Ndenga et al. (2016), who reported the total annual EIRs as 31.1, 16.6, and 0.4 ib/p/yr at Kombewa, Iguhu, and Marani, respectively [23]. In the current study, the lower inoculation rates recorded could be attributed to reduced vector densities and, to some extent, a shift to non-human feeding by the malaria vectors due to high bednet coverage in the study areas [54]. Nevertheless, *An*. *funestus* and *An*. *gambiae* s.l. played major roles in malaria transmission in Kombewa and Iguhu, respectively, despite the comparatively low vector densities, indicating high vectorial efficiency of these anophelines in transmitting malaria in the region.

One limitation of our study was that parasitological surveys were based on microscopy only, which may not detect light plasmodial infections compared to highly sensitive PCR-based techniques. Hence, the *P*. *falciparum* prevalence and infection pattern may have been underestimated. A second limitation was the lack of long-term information on outdoor malaria transmission dynamics, which may have provided insight to the resurgence in *P*. *falciparum* transmission despite continuous intervention efforts.

## Conclusions

Malaria prevalence remains high and has resurged in some sites in western Kenya despite continuous intervention efforts. Hence, long-time monitoring of malaria transmission profiles is essential in evaluating the success of current interventions, accurately measuring changing malaria epidemiology, and directing strategies for future control and elimination efforts. Residing in malaria-endemic villages and male gender were significant risk factors associated with asymptomatic malaria infection, with these individuals serving as human reservoirs for sustained malaria transmission. Consequently, targeted control might effectively reduce those with asymptomatic infections and potentially decrease malaria transmission and prevent resurgences.

## Supplementary Information

**Additional file 1: Figure S1.** Distribution of the proportion of surveys being infected in Kombewa (**a**), Iguhu (**b**), and Marani (**c**) in western Kenya.

**Additional file 2: Figure S2.** Heat map showing the *Plasmodium falciparum* infection patterns in Kombewa, Iguhu, and Marani in western Kenya.

**Additional file 3: Table S1.** Malaria infection in Kombewa, Iguhu, and Marani in western Kenya [Mean (95%CI)].

**Additional file 4: Table S2.** Hazard ratios for the infection in Kombewa, Iguhu, and Marani in western Kenya.

**Additional file 5: Table S3.** Host feeding preference of *Anopheles* mosquitoes in Kombewa, Iguhu, and Marani in western Kenya.

**Additional file 6: Figure S3.** Variations in monthly maximum temperature, minimum temperature, mean temperature and monthly rainfalls in Kombewa (**a**), Iguhu (**b**), and Marani (**c**) in western Kenya.

## Data Availability

All data generated or analyzed during this study are included in this published article and its additional files.

## Abbreviations

ACTs: Artemisinin-based combination therapies
DNA: Deoxyribonucleic acid
EIRs: Entomological inoculation rates
HBI: Human biting index
ICEMR: International Center of Excellence for Malaria Research
IRS: Indoor residual spraying
ITNs: Insecticide-treated nets
LLINs: Long-lasting insecticidal nets
PBO: Piperonyl butoxide
PCR: Polymerase chain reaction
PSC: Pyrethrum spray catches
qPCR: Quantitative polymerase chain reaction
s.l.: sensu lato
s.s.: sensu stricto
WHO: World Health Organization
