# Supplementary figures and images for: A prospective cohort study of Plasmodium falciparum malaria in three sites of Western Kenya
Source: Parasit Vectors. 2022 Nov 9;15:416. doi: 10.1186/s13071-022-05503-4 (PMC9647947; doi:10.1186/s13071-022-05503-4)

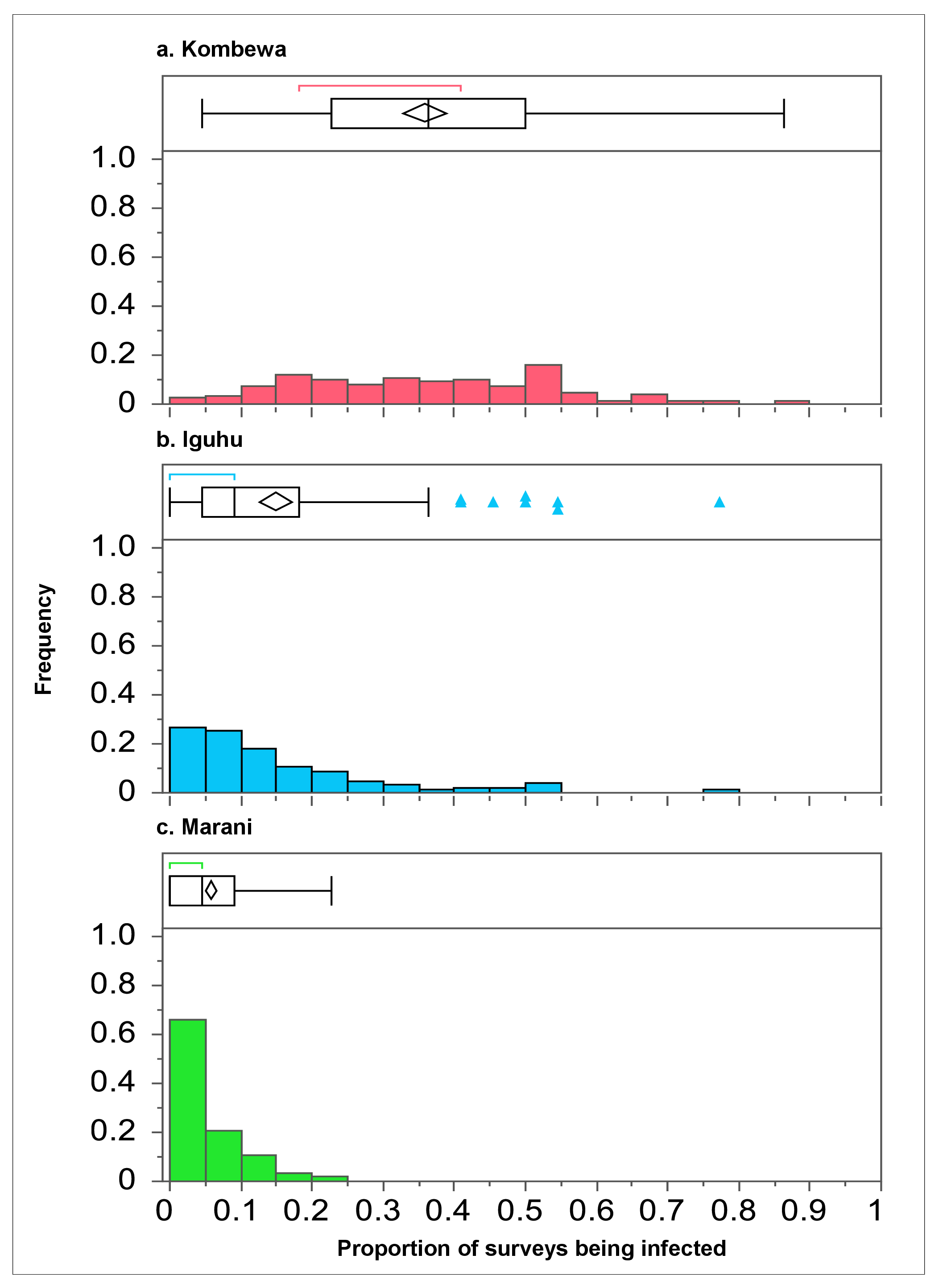

Supplement: Supplementary file 1 — Additional file 1: Figure S1. Distribution of the proportion of surveys being infected in Kombewa (a), Iguhu (b), and Marani (c) in western Kenya. [file 13071_2022_5503_MOESM1_ESM.tiff]

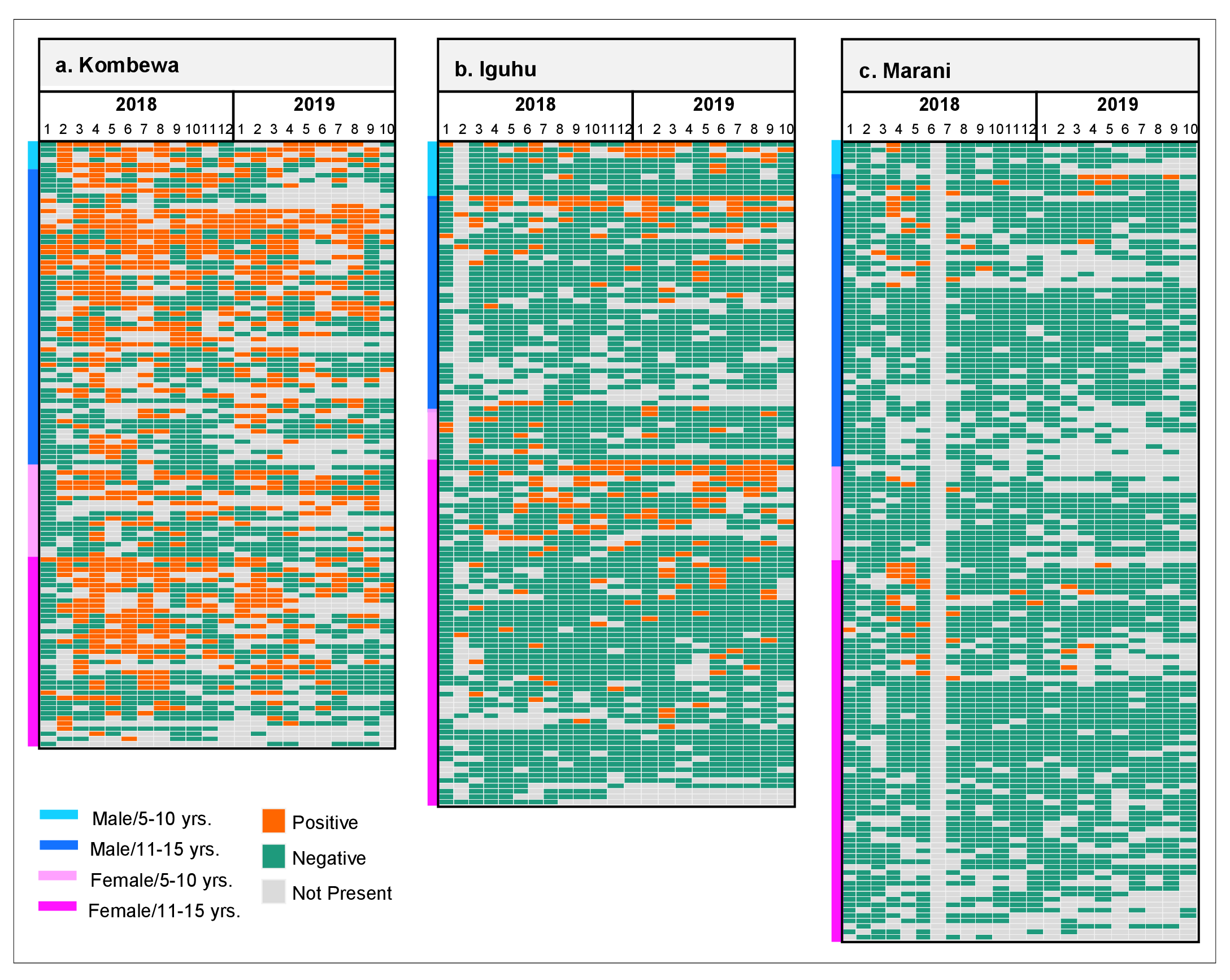

Supplement: Supplementary file 2 — Additional file 2: Figure S2. Heat map showing the Plasmodium falciparum infection patterns in Kombewa, Iguhu, and Marani in western Kenya. [file 13071_2022_5503_MOESM2_ESM.tiff]

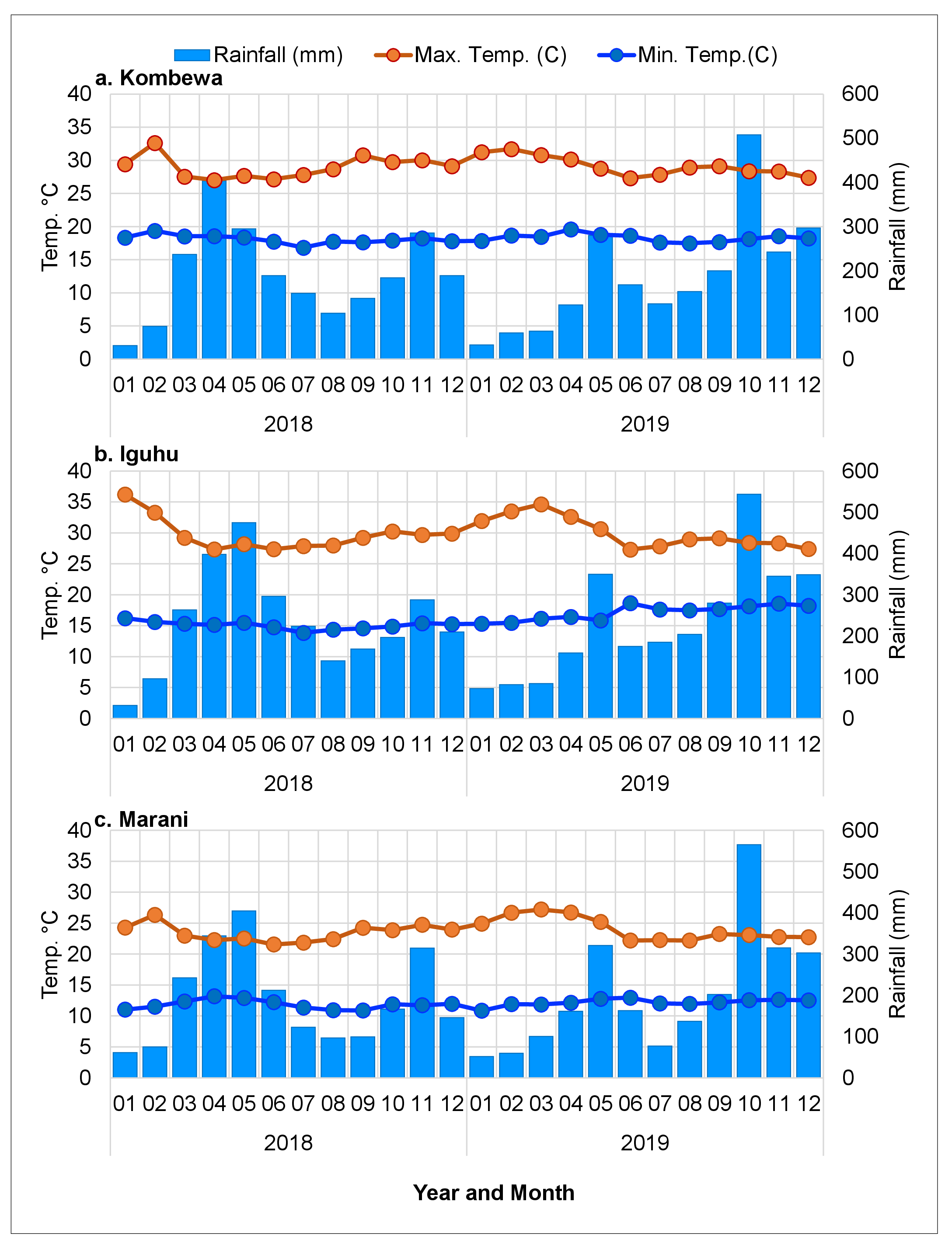

Supplement: Supplementary file 6 — Additional file 6: Figure S3. Variations in monthly maximum temperature, minimum temperature, mean temperature and monthly rainfalls in Kombewa (a), Iguhu (b), and Marani (c) in western Kenya. [file 13071_2022_5503_MOESM6_ESM.tiff]
