# Supplementary material for: A prospective cohort study of Plasmodium falciparum malaria in three sites of Western Kenya
Source: Parasit Vectors. 2022 Nov 9;15:416. doi: 10.1186/s13071-022-05503-4 (PMC9647947; doi:10.1186/s13071-022-05503-4)
Supplement: Supplementary file 3 — Additional file 3: Table S1. Malaria infection in Kombewa, Iguhu, and Marani in western Kenya [Mean (95%CI)]. [file 13071_2022_5503_MOESM3_ESM.docx]

**Additional file 3: Table S1** Malaria infection in Kombewa, Iguhu, and Marani in western Kenya [Mean (95%CI)]

| **Sites** |  |  | **No. Samples^a^** | **Mean Parasite Prevalence (%)** | ***P*^b^** | **Mean Parasite Density (/µl)^c^** | ***P*^d^** | **Proportion of Months Infected (%)^e^** | ***P*^d^** |
| --- | --- | --- | --- | --- | --- | --- | --- | --- | --- |
| **Kombewa** | **Age** | 5-10 yrs | 734 | 45.23  (41.63, 48.83) | χ^2^=2.88, *d.f.*=1, *P*>0.05 | 1836.27  (1359.46, 2313.07) | Z=4.49, *P*<0.0001 | 35.61  (29.41, 41.80) | Z=0.16,  *P*>0.05 |
|  |  | 11-15 yrs | 1,408 | 41.41  (38.83, 43.98) |  | 1218.25  (894.33, 1542.17) |  | 36.00  (32.35, 39.65) |  |
|  | **Gender** | Female | 1,036 | 36.97  (34.03, 39.91) | χ^2^=27.17, *d.f.*=1, *P*<0.0001 | 1330.03  (929.91, 1730.14) | Z=3.32, *P*<0.001 | 32.83  (28.44, 37.22) | Z=1.65,  *P*>0.05 |
|  |  | Male | 1,106 | 48.10  (45.16, 51.05) |  | 1523.46  (1159.46, 1887.45) |  | 38.49  (34.14, 42.85) |  |
| **Iguhu** | **Age** | 5-10 yrs | 620 | 14.52  (11.74, 17.29) | χ^2^=2.47, *d.f.*=1, *P*>0.05 | 2290.11  (698.57, 1657.88) | Z=1.65, *P*>0.05 | 15.80  (9.24, 22.36) | Z=0.09,  *P*>0.05 |
|  |  | 11-15 yrs | 2,011 | 12.08  (10.66, 13.51) |  | 2948.64  (963.01, 4934.27) |  | 14.71  (12.16, 17.25) |  |
|  | **Gender** | Female | 1,669 | 12.28  (10.71, 13.86) | χ^2^=0.57, *d.f.*=1, *P*>0.05 | 2826.15  (703.63, 4948.67) | Z=0.41, *P*>0.05 | 14.06  (11.37, 16.74) | Z=0.58  *P*>0.05 |
|  |  | Male | 962 | 13.31  (11.16, 15.45) |  | 1900.00  (207.32, 3592.68) |  | 16.19  (11.76, 20.62) |  |
| **Marani** | **Age** | 5-10 yrs | 592 | 2.53  (1.27, 3.80) | χ^2^=0.15, *d.f.*=1, *P*>0.05 | 288.00  (33.06, 542.94) | Z=0.69, *P*>0.05 | 3.93  (1.93, 5.92) | Z=1.93  *P*>0.05 |
|  |  | 11-15 yrs | 1,980 | 2.83  (2.10, 3.56) |  | 349.29  (24.27, 674.30) |  | 6.10  (5.18, 7.01) |  |
|  | **Gender** | Female | 1,541 | 2.73  (1.91, 3.54) | χ^2^=0.02, *d.f.*=1, *P*>0.05 | 235.24  (122.29, 348.19) | Z=1.26, *P*>0.05 | 5.55  (4.50, 6.60) | Z=0.46  *P*>0.05 |
|  |  | Male | 1,031 | 2.81  (1.80, 3.82) |  | 482.76  (0, 1115.95) |  | 6.09  (4.70, 7.47) |  |

^a^ No. samples referred to the total number of data points used for the calculation in the repeated sampling scheme.

^b^ *P* values were calculated with Chi-square tests.

^c^ Geometric mean was used.

^d^ *P* values were calculated with Wilcoxon/Kruskal-Wallis tests.

^e^ Individuals enrolled between January to March 2018, with at least 6 tests done in the cohort were used.
