# Supplementary material for: A prospective cohort study of Plasmodium falciparum malaria in three sites of Western Kenya
Source: Parasit Vectors. 2022 Nov 9;15:416. doi: 10.1186/s13071-022-05503-4 (PMC9647947; doi:10.1186/s13071-022-05503-4)
Supplement: Supplementary file 4 — Additional file 4: Table S2. Hazard ratios for the infection in Kombewa, Iguhu, and Marani in western Kenya. [file 13071_2022_5503_MOESM4_ESM.docx]

**Additional file 4: Table S2** Hazard ratios for the infection in Kombewa, Iguhu, and Marani in western Kenya

| **Predictors** | | **Hazard ratio (95% CI^a^)** | | | | |
| --- | --- | --- | --- | --- | --- | --- |
|  |  | **Unadjusted** | ***P*^b^** |  | **Adjusted** | ***P*^b^** |
| **Sites** | **Marani** | ref |  |  | ref |  |
|  | **Kombewa** | 6.22 (5.32, 7.27) | <0.0001 |  | 6.12 (5.22, 7.16) | <0.0001 |
|  | **Iguhu** | 2.58 (2.17, 3.07) | <0.0001 |  | 2.59 (2.18, 3.08) | <0.0001 |
| **Gender** | **Male** | ref |  |  | ref |  |
|  | **Female** | 0.74 (0.67, 0.82) | <0.0001 |  | 0.97 (0.89, 1.02) | 0.005 |
| **Age** | **5-10 yrs** | ref |  |  | ref |  |
|  | **11-15 yrs** | 0.84 (0.75, 0.95) | 0.007 |  | 1.01 (0.90, 1.15) | 0.84 |

^a^ Wald approximations were used for ratio confidence limits effects.

^b^ *P* values were calculated with proportional hazards fit.
