# Supplementary material for: A prospective cohort study of Plasmodium falciparum malaria in three sites of Western Kenya
Source: Parasit Vectors. 2022 Nov 9;15:416. doi: 10.1186/s13071-022-05503-4 (PMC9647947; doi:10.1186/s13071-022-05503-4)
Supplement: Supplementary file 5 — Additional file 5: Table S3. Host feeding preference of Anopheles mosquitoes in Kombewa, Iguhu, and Marani in western Kenya. [file 13071_2022_5503_MOESM5_ESM.docx]

**Additional file 5: Table S3** Host feeding preference of *Anopheles* mosquitoes in Kombewa, Iguhu, and Marani in western Kenya

| **Study sites and blood-meal origins** | **Kombewa** | |  | **Iguhu** | |  | **Marani** | |
| --- | --- | --- | --- | --- | --- | --- | --- | --- |
|  | ***An*. *gambiae* s.l. (%)** | ***An*. *funestus* (%)** |  | ***An*. *gambiae* s.l. (%)** | ***An*. *funestus* (%)** |  | ***An*. *gambiae s.l.* (%)** | ***An*. *funestus* (%)** |
| No. tested | 63 | 59 |  | 87 | 35 |  | 1 | 6 |
| Human | 18 (28.57) | 44 (74.58) |  | 29 (33.33) | 23 (65.71) |  | 1 (100.00) | 2 (33.33) |
| Bovine | 29 (46.03) | 4 (6.78) |  | 45 (51.72 | 1 (2.86) |  | 0 (0.00) | 3 (50.00) |
| Goat | 0 (0.00) | 1 (1.69) |  | 1 (1.15) | 0 (0.00) |  | 0 (0.00) | 0 (0.00) |
| Pig | 1 (1.59) | 2 (3.39) |  | 0 (0.00) | 0 (0.00) |  | 0 (0.00) | 0 (0.00) |
| Dog | 1 (1.59) | 0 (0.00) |  | 0 (0.00) | 0 (0.00) |  | 0 (0.00) | 0 (0.00) |
| Human + bovine | 5 (7.93) | 0 (0.00) |  | 2 (2.30) | 8 (22.86) |  | 0 (0.00) | 0 (0.00) |
| Human + dog | 0 (0.00) | 4 (6.78) |  | 0 (0.00) | 0 (0.00) |  | 0 (0.00) | 0 (0.00) |
| Human + pig | 0 (0.00) | 1 (1.69) |  | 0 (0.00) | 2 (5.71) |  | 0 (0.00) | 1 (16.67) |
| Human + goat | 4 (6.35) | 2 (3.39) |  | 2 (2.30) | 1 (2.86) |  | 0 (0.00) | 0 (0.00) |
| Human + bovine + pig + goat | 0 (0.00) | 0 (0.00) |  | 1 (1.15) | 0 (0.00) |  | 0 (0.00) | 0 (0.00) |
| Bovine + goat | 1 (1.59) | 0 (0.00) |  | 0 (0.00) | 0 (0.00) |  | 0 (0.00) | 0 (0.00) |
| Pig + goat | 1 (1.59) | 0 (0.00) |  | 0 (0.00) | 0 (0.00) |  | 0 (0.00) | 0 (0.00) |
| Unknown | 3 (4.76) | 1 (1.69) |  | 7 (8.05) | 0 (0.00) |  | 0 (0.00) | 0 (0.00) |
| **HBI^a^** | **42.86%** | **86.44%** |  | **39.08%** | **97.14%** |  | **100.00%** | **50.00%** |

^a^ Human blood index (HBI) was calculated as the number of mosquitoes positive for human blood-meal (including mixed blood-meal) divided by the total number tested.
